# Supplementary material for: Quantitative contributions of TNF receptor superfamily members to CD8+ T‐cell responses
Source: Mol Syst Biol. 2021 Nov 22;17(11):e10560. doi: 10.15252/msb.202110560 (PMC8607805; doi:10.15252/msb.202110560)
Supplement: Supplementary file 1 — Appendix [file MSB-17-e10560-s001.pdf]

Appendix

Contents

1 Appendix Table 2
2 Appendix Figures 3

1 | APPENDIX TABLE

APPENDIX TABLE S1 Parameters for operational model

Parameter	Values in Fig. 4 (Model)	Values in Fig. 5 (Synergy)
KD = 1/KA	$10^{2.2}$	$10^{2.2}$
n	1.35	1.35
k1	$10^{-4.5}$	$10^{-4.5}$
k2	$10^{(} - 3.0)$	$10^{-3.0}$
k3 (for cytokine)	$10^{-4.0}$	$10^{-4.0}$
μ	$10^{-1.0}$	$10^{-1.0}$
σ	$10^{-1.8}$	$10^{-1.8}$
k4	$10^{-4.8}$	$10^{-4.6}$
CD70	0 or 200	0 or 200
KdCD27	$10^{1.5}$	$10^{1.5}$
k5	$10^{-6.0}$	$10^{-6.0}$
k6	$10^{-3.7}$	$10^{-3.7}$
41BBL	0 or 500	0 or 500
Kd41BB	$10^{2.3}$	$10^{2.3}$
k3 (for 41BB)	$10^{-4.4}$	$10^{-4.4}$
k7	$10^{-8.0}$	$10^{-8.0}$
k8	$10^{-4.0}$	$10^{-4.0}$
k9	$10^{-5.0}$	$10^{-5.0}$
k10 (for cytokine)	$10^{0.8}$	$10^{0.8}$
k11 (for cytokine)	$10^{1.5}$	$10^{1.2}$
k10 (for 41BB)	$10^{0.5}$	$10^{0.5}$
k11 (for 41BB)	$10^{0.5}$	$10^{0.5}$

2 | APPENDIX FIGURES

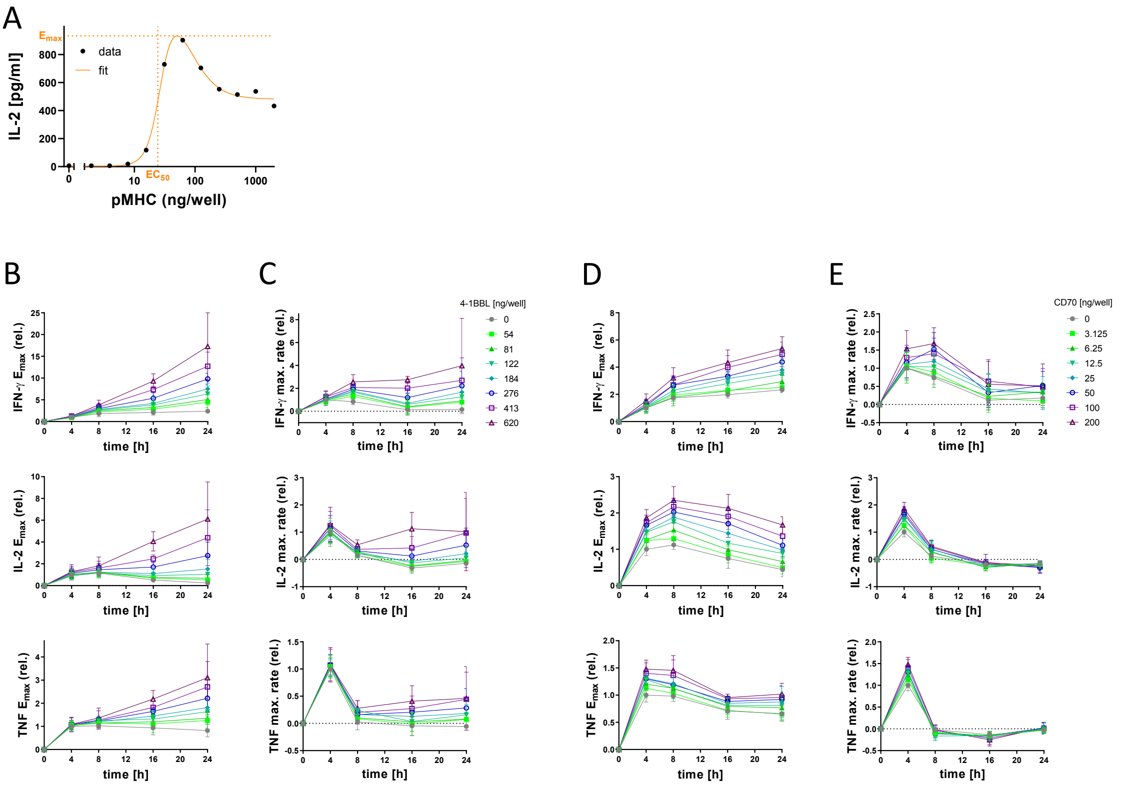

APPENDIX FIGURE S1 4-1BB co-stimulation delays adaptation of the T cell cytokine response, whereas CD27 co-stimulation only increases early cytokine production. Primary human CD8+ T cells transduced with the c58/c61 TCR were stimulated for 4, 8, 16 and 24 hours with plate-immobilised pMHC, with or without 4-1BBL in (B) and (C) or CD70 in (D) and (E) at the indicated doses. The production of the cytokines IFN-γ, IL-2 and TNF into the culture medium supernatant was quantified by ELISA. **(A)** Cytokine dose-response curve fit and extraction of metrics. As an example, an IL-2 dose-response data set was fit with a bell-shaped function as described in the Methods. Graphically shown as dotted lines are the E_{max} and EC_{50} which were empirically determined. In **(B)** and **(D)**, cytokine E_{max} values from three independent experiments were extracted from dose-response curve fits and normalised to the amount of cytokine produced without co-stimulation at the 4 h time point. In **(C)** and **(E)**, average rates of cytokine production were calculated from the extracted E_{max} values and normalised to the rate of cytokine production in the first 4 hours without co-stimulation.

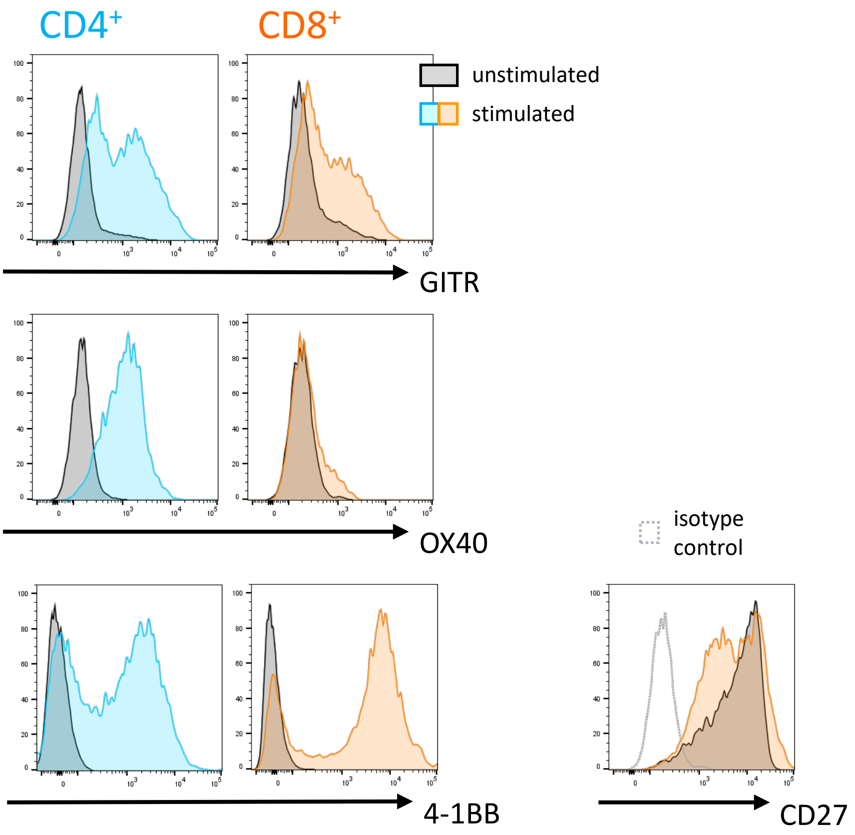

APPENDIX FIGURE S2 GITR and OX40 expression is induced on CD8$^+$ T cells to a lesser extent than on CD4$^+$ T cells. Expanded primary human CD4$^+$ and CD8$^+$ T cell blasts (day 10-13) were stimulated with plate-immobilised anti-CD3 antibodies (clone UCHT1, 1µg/ml) for 18 hours. The indicated surface receptors were labelled with fluorescent antibodies and analysed by flow cytometry. Shown here are flow cytometry histograms for unstimulated (grey) and stimulated (blue/orange) T cells.

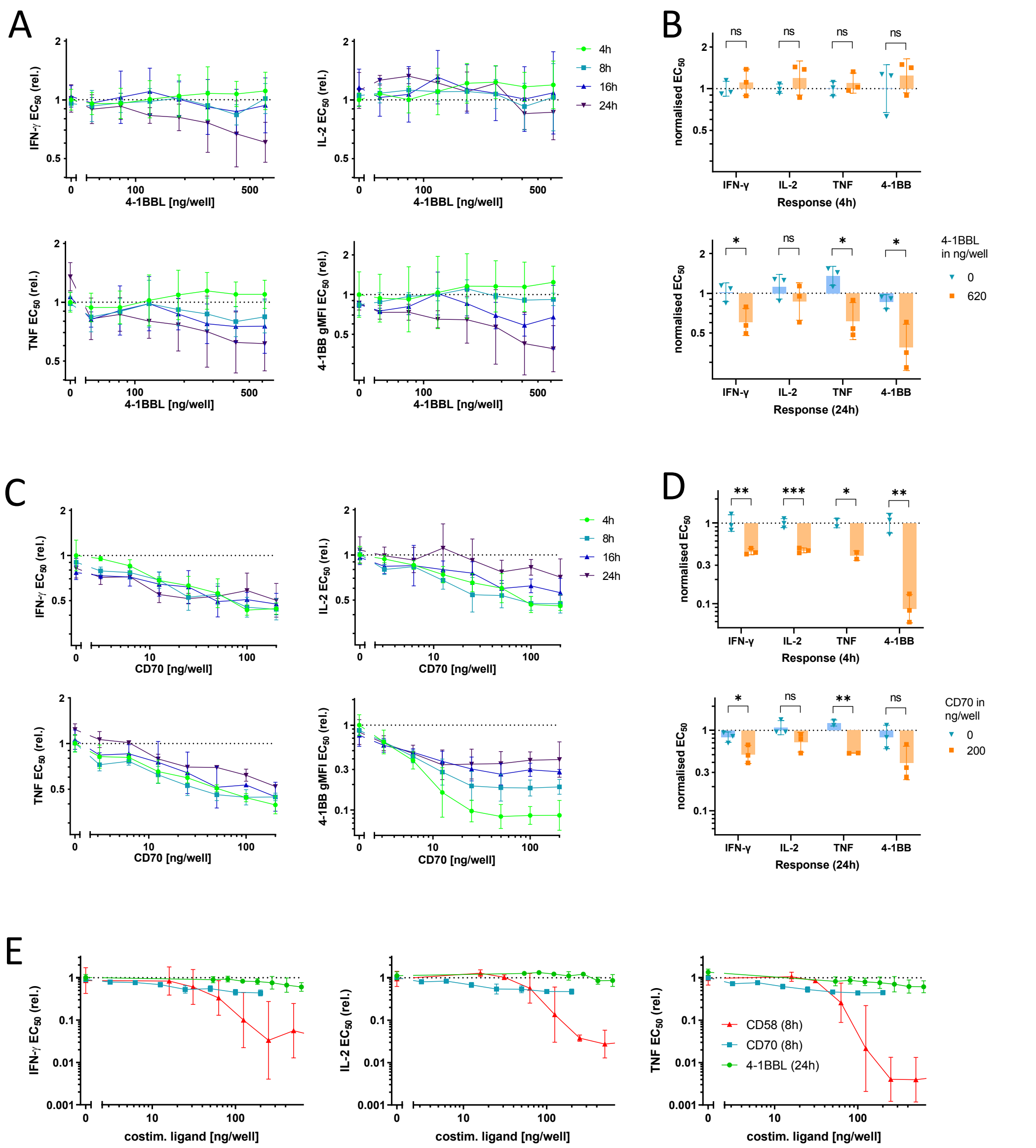

APPENDIX FIGURE S3 Co-stimulation through TNFRSF members lowers the EC_{50} of the T cell response to pMHC. Primary human $CD8^{+}$ T cells transduced with the c58/c61 TCR were stimulated for 4, 8, 16 and 24 hours with plate-immobilised pMHC, with or without 4-1BBL in (A) and (B) or CD70 in (C) and (D) at the indicated doses. The production of the cytokines IFN-γ, IL-2 and TNF into the culture medium supernatant was quantified by ELISA. Surface 4-1BB was labelled with a fluorescent anti-4-1BB antibody and quantified by flow cytometry. In **(A)** and **(C)**, EC_{50} values from three separate experimental repeats were extracted from dose-response curve fits and normalised to the 4-hour time point without co-stimulation. To quantify statistically significant effects on sensitivity, EC_{50} values at 4 or 24 hours were compared in **(B)** and **(D)** with two-tailed t-tests between the conditions with or without co-stimulation. For comparison, the same experiment was carried out using CD58 as co-stimulatory ligand to CD2 **(E)**. EC_{50} values from three separate experimental repeats were extracted from dose-response curve fits, normalised to the 4-hour time point without co-stimulation and plotted along with selected data from (A) and (C). Abbreviations: ns (not significant) = p-value > 0.05; * = p-value < 0.05

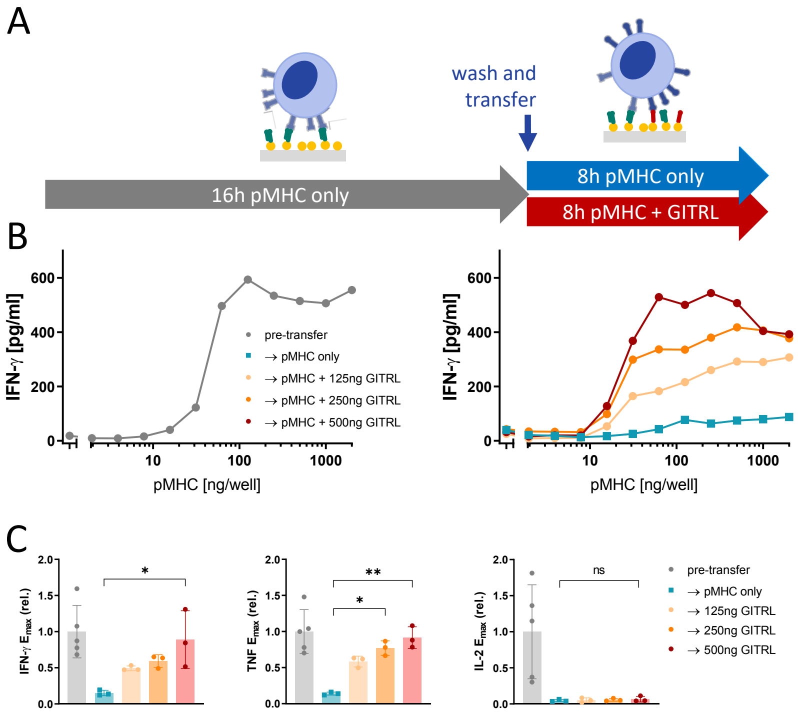

APPENDIX FIGURE S4 GITR co-stimulation is capable of partially rescuing the cytokine response in already adapted T cells. (A) Schematic of the experimental design for (B) and (C). Primary human CD8$^+$ T cells transduced with the c58/c61 TCR were stimulated for 16h with pMHC doses varying from 0 to 2000 ng/well. Cells were harvested, washed and stimulated for further 8h with identical pMHC doses which they were adapted to, in absence or presence of GITRL. The production of the cytokines IFN-γ, IL-2 and TNF into the culture medium supernatant was quantified by ELISA. **(B)** T cell response during the first 16h stimulation from one representative experiment (left) and during the secondary 8h stimulation from the same experiment (right). **(C)** E_{max} values from three separate experimental repeats were extracted from dose-response curve fits and normalised to the cytokine response during the 16h pre-stimulation. Post-transfer conditions were compared using one-way ANOVA with Šídák's correction for multiple comparisons. Abbreviations: ns = p-value > 0.05; * = p-value < 0.05; ** = p-value < 0.01

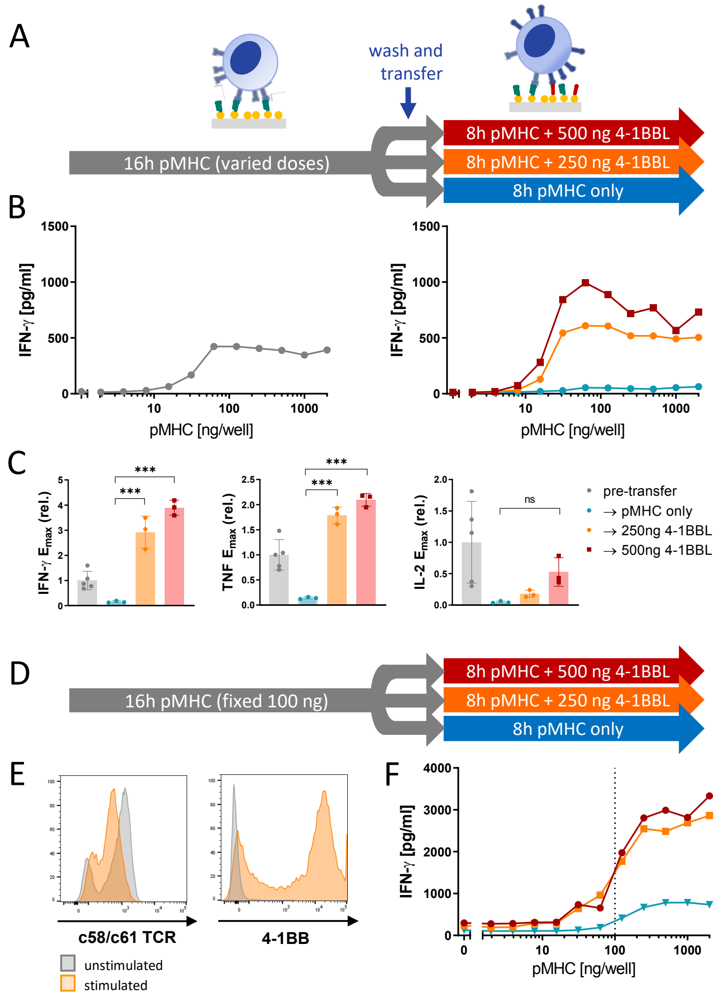

APPENDIX FIGURE S5 4-1BB co-stimulation rescues the cytokine response in already adapted T cells in a TCR-signalling-dependent manner. (A) Schematic of the experimental design. Primary human CD8^{+} T cells transduced with the c58/c61 TCR were stimulated for 16h with pMHC doses varying from 0 to 2000 ng/well. Cells were harvested, washed and stimulated for further 8h with identical pMHC doses which they were adapted to, in absence or presence of 4-1BBL. The production of the cytokines IFN-γ, IL-2 and TNF into the culture medium supernatant was quantified by ELISA. **(B)** T cell response during the first 16h stimulation from one representative experiment (left) and during the secondary 8h stimulation from the same experiment (right). **(C)** E_{max} values from three separate experimental repeats were extracted from dose-response curve fits and normalised to the cytokine response during the 16h pre-stimulation. Post-transfer conditions were compared using one-way ANOVA with Šídák's correction for multiple comparisons. **(D)** For (E) and (F), T cells were uniformly stimulated for 16h with pMHC and CD70 (each at 100 ng/well) in order to induce 4-1BB expression with minimal TCR downregulation. Cells were harvested, washed, and stimulated for a further 8h with pMHC and 4-1BBL at the indicated doses. **(E)** TCR and 4-1BB expression on cells before and after the 16h pre-stimulation from one representative experiment out of four. **(F)** T cell response during the secondary 8h stimulation from one representative experiment out of four. The production of the cytokines IFN-γ, IL-2 and TNF into the culture medium supernatant was quantified by ELISA. Abbreviations: ns = p-value > 0.05; * = p-value < 0.05; ** = p-value < 0.01; *** = p-value < 0.001.

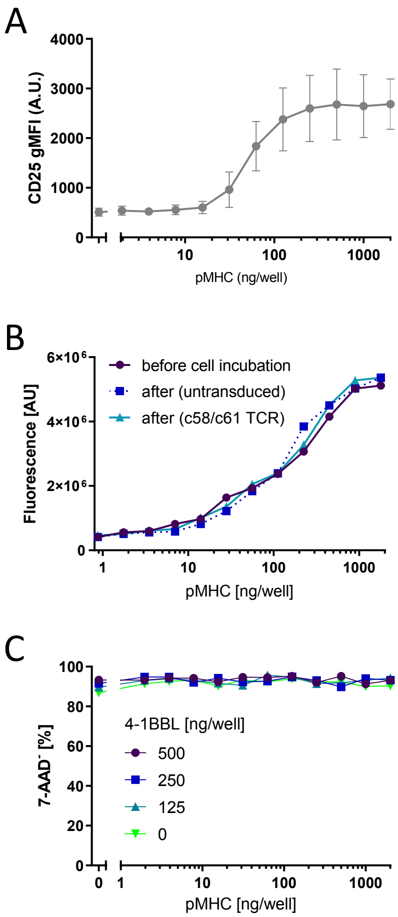

APPENDIX FIGURE S6 Testing limitations of the plate-based experimental system. (A) T cell activation induces the expression of CD25, the IL-2 receptor α-chain. Primary human $CD8^+$ T cells transduced with the c58/c61 TCR were stimulated for 16 hours with pMHC at the indicated doses. Surface CD25 was labelled with a fluorescent anti-CD25 antibody and quantified by flow cytometry. Shown here is the averaged dose-response curve for 3 experimental repeats. **(B)** Harvesting cells from coated plates after stimulation does not dislodge detectable amounts of plate-bound pMHC. Densities of pMHC immobilised on streptavidin-coated plates were quantified using a conformation-sensitive anti-HLA-A/B/C antibody (clone w6/32) either before or after incubation with primary human $CD8^+$ T cells (either transduced with the c58/c61 TCR or untransduced) for 16h. **(C)** Transfer of cells between stimulation plates does not cause notable levels of cell death. Primary human $CD8^+$ T cells transduced with the c58/c61 TCR were stimulated for 16h with pMHC and CD70 (each at 100 ng/well). Cells were harvested, washed, and stimulated for further 8h with pMHC and 4-1BBL at the indicated doses. Cell death after the experiment was quantified by flow cytometry using 7-AAD staining.

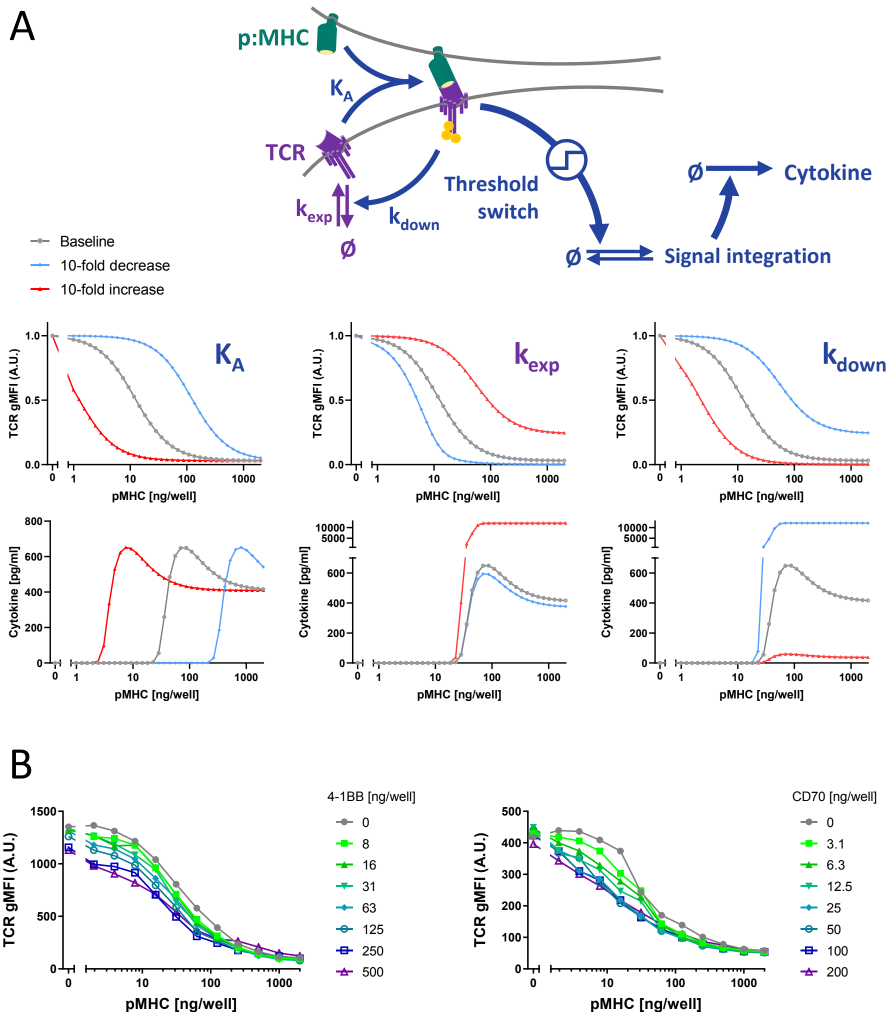

APPENDIX FIGURE S7 TNFRSF co-stimulation phenotypes cannot be simulated by modulation of TCR expression, downregulation or avidity to pMHC. As in the base model of Figure 4, T cell receptor (TCR) and peptide-major histocompatibility complex (pMHC) form a receptor-ligand complex that induces the cytokine response, gated by a threshold switch. At the same time, ligand binding causes downregulation of the TCR. **(A)** Potential effects of modulation of TCR-pMHC association constant (K_A), TCR expression rate (k_{exp}) and the rate of induced TCR downregulation (k_{down}) by co-stimulation on TCR surface expression and cytokine production were simulated as 10-fold increases or decreases of the respective parameters. **(B)** Primary human CD8$^+$ T cells transduced with the c58/c61 TCR were stimulated for 8 hours with plate-immobilised pMHC and ligands to TNFRSF members at the indicated doses. Surface TCR was labelled with fluorescent pMHC tetramers and quantified by flow cytometry.

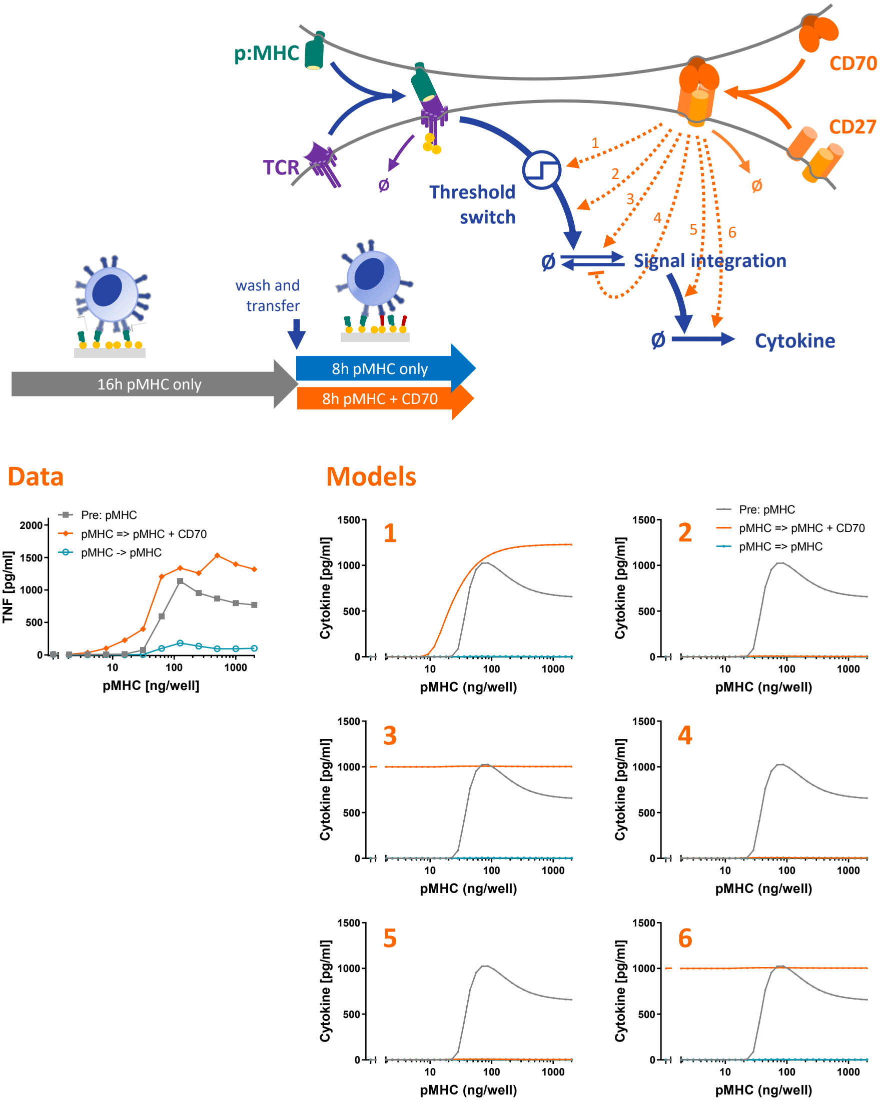

APPENDIX FIGURE S8 The effect of TNFRSF co-stimulation on the T cell response in transfer experiments can be explained by modulation of the activation threshold. The model in Fig. 4 is used to simulate the transfer experiment (see Data) for 6 different integration points for CD27 co-stimulation. As observed in the edxperimental data, a pMHC-dependent T cell response in the second stimulation (orange curves) is only observed in model 1 when CD27 lowers the activation threshold of the switch.

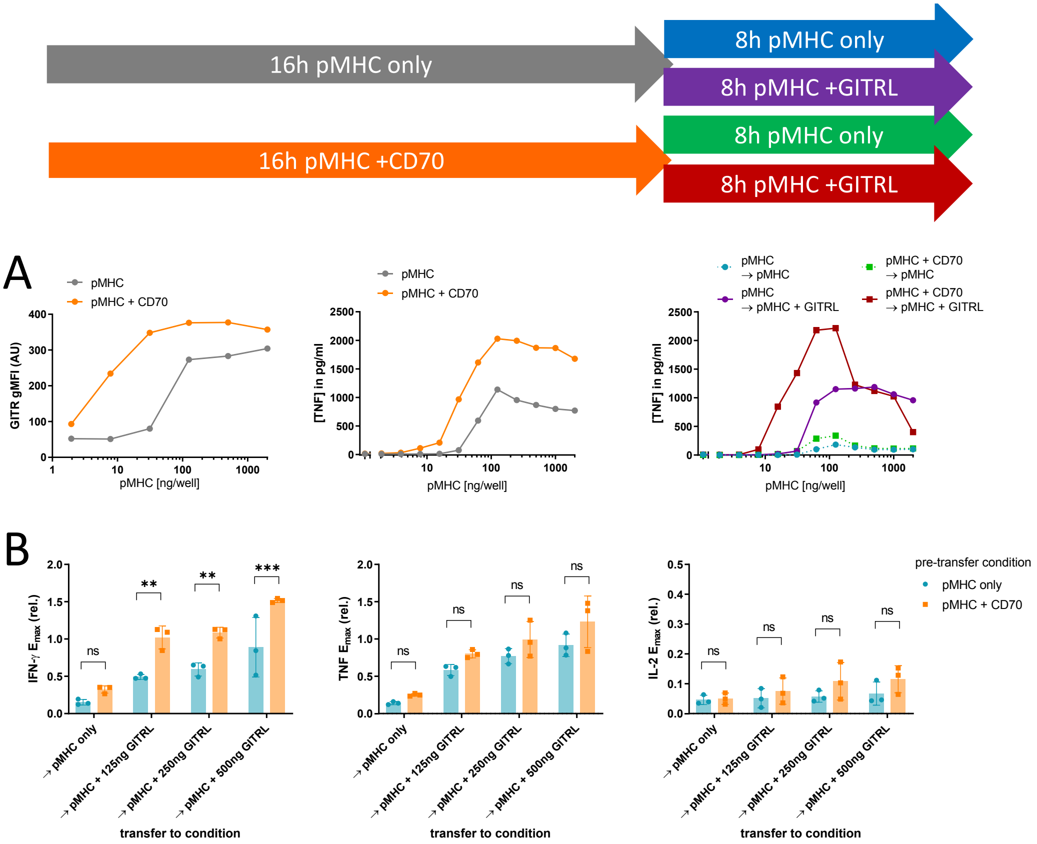

APPENDIX FIGURE S9 Co-stimulation through CD27 and GITR synergise. Primary human CD8+ T cells transduced with the c58/c61 TCR were stimulated for 16h with pMHC doses varying from 0 to 2000 ng/well in presence or absence of 200 ng/well CD70. Cells were harvested, washed and stimulated for further 8h with identical pMHC doses which they were adapted to, with or without addition of 500 ng/well GITRL. The production of the cytokines IFN-γ, IL-2 and TNF into the culture medium supernatant was quantified by ELISA. **(A)** T cell response during the first 16h stimulation from one representative experiment (middle) and during the secondary 8h stimulation from the same experiment (right). Cells from designated duplicate samples in the same experiment were stained with fluorescent anti-GITR antibodies after the first 16h stimulation and analysed by flow cytometry (left). **(B)** E_{max} values from three separate repeats of the experiment were extracted from dose-response curve fits and normalised to the cytokine response without co-stimulation during the 16h pre-stimulation. Post-transfer conditions in were compared using one-way ANOVA with Šídák's correction for multiple comparisons. Abbreviations: ns (not significant) = p-value > 0.05; * = p-value < 0.05; ** = p-value < 0.01; *** = p-value < 0.001
