# Supplementary material for: Quantitative contributions of TNF receptor superfamily members to CD8+ T‐cell responses
Source: Mol Syst Biol. 2021 Nov 22;17(11):e10560. doi: 10.15252/msb.202110560 (PMC8607805; doi:10.15252/msb.202110560)
Supplement: Supplementary file 3 [file MSB-17-e10560-s002.pdf]

molecular systems biology

Quantitative contributions of TNF receptor superfamily members to CD8$^+$ T cell responses

John Nguyen, Johannes Pettmann, Philipp Kruger, and Omer Dushek
DOI: 10.15252/msb.202110560

Corresponding author(s): Omer Dushek (omer.dushek@path.ox.ac.uk)

Review Timeline:

Submission Date:	7th Jul 21
Editorial Decision:	6th Sep 21
Revision Received:	29th Sep 21
Editorial Decision:	12th Oct 21
Revision Received:	22nd Oct 21
Accepted:	25th Oct 21

Editor: Jingyi Hou

Transaction Report:

(Note: With the exception of the correction of typographical or spelling errors that could be a source of ambiguity, letters and reports are not edited. Depending on transfer agreements, referee reports obtained elsewhere may or may not be included in this compilation. Referee reports are anonymous unless the Referee chooses to sign their reports.)

Thank you for submitting your work to Molecular Systems Biology. We are still expecting a report from Reviewer #2, but since the recommendations of the other two reviewers are quite similar, I prefer to make a decision now rather than further delaying the process. If we receive comments from Reviewer #2, we will forward them to you so that you can address any further issues raised. As you will see from the reports below, the reviewers acknowledge the potential interest of the study. They raise however a series of concerns, which we would ask you to address in a major revision.

Since the reviewers' recommendations are rather clear, there is no need to reiterate all the points listed below. All issues raised by the reviewers need to be satisfactorily addressed. As you may already know, our editorial policy allows in principle a single round of major revision and it is therefore essential to provide responses to the reviewers' comments that are as complete as possible.

On a more editorial level, we would ask you to address the following issues.

REFEREE REPORTS

Reviewer #1:

This is a polished paper with some strong data that support conclusions based on an elegant yet simple and conceptually compelling analysis. I am not a T-cell biologist (and cannot comment on the T-cell biology relevance of the work) but II found the work elegant and inspiring. The model is coarse, described as operational. It would be nice to connect it with more detailed TCR math models and hence identify the molecular mechanisms by which the co-stimulatory receptors have the effect they do. But for a short paper in MSB this may be outside the scope. The elegance of the current scope is compelling for a short paper.

Reviewer #3:

In this manuscript, Nyugen et al combine experimental and modeling approaches to quantitatively investigate how costimulatory signaling in T cells is integrated with TCR activation. The authors use plate-bound ligand presentation to modulate the T cell input over a range of different parameters, using approaches that are well established in the Dushek lab. Through this work, the authors explore the differential inputs that the TNF receptor superfamily provide. They find that while GITR and OX40 have minimal influence on cytokine secretion, CD27 and 4-1BB can provide substantial co-stimulation to CD8+ T cells, with 4-1BB capable of prolonging cytokine production. They use mathematical modeling to predict that this result is driven by continued 4-1BB gene expression, which they then demonstrate through further experiments. Their "take-home" message is that for the TNFR family, each receptor can have quantitatively distinct functions based on level of expression and position of integration within signalling network.

Overall, the work presented was performed well, the main aim was clearly defined and the conclusions the authors draw are

interesting and convincing. While there has been much previous work on costimulatory receptors in T cell activation, it has primarily remained on the qualitative scale, with little nuance about the relative dynamics of this secondary input over the course of T cell activation. The advance presented in this manuscript is that different members of TNFR family of costimulatory receptors provide 'help' at different stages of T cell activation, and the intracellular wiring allows specific receptors to maintain their expression to prolong the length as well as strength of this secondary input. The audience for this manuscript would be those working in T cell signaling, and also in CAR-T function, where the signaling motifs of some of the TNFRSF members are used within this context. The authors also allude to this in their discussion. The iterative use of experimental and modeling approaches would also be of interest to those more broadly working in other signaling systems.

I provide some points below that I hope will improve the manuscript for the broader MSB reader, as well as help reinforce some of their conclusions.

Major points

1. Why were CD8+ T cells, and not CD4+ T cells used for these experiments, or even both? Co-stimulation is important for both T cell subsets. The differences between these subtypes were also not defined, which would be helpful for the general MSB reader. It would be good to explicitly state why the authors thought CD8+ T cells were the better choice for these experiments. Would they expect similar results for CD4+ T cells?

2. Following on from this, what state were activated/expanded CD8+ T cells in before plate-bound assays? How would this compare to naïve T cells, say? I think it is important to confirm what phenotype they had given that CTL, for instance, have minimal requirement for co-stimulation.

3. Page 1, Para 3: "Operational" needs to be defined explicitly within text, given its continued use when describing models later. The reference doesn't really help here.

4. Page 4, para 2: It was quite hard to visualise the "burst of cytokine" when there isn't a time course to directly view this in Fig 2B. To me, it looked like for GITR and OX40 IFNg is increasing with time. Maybe this is just noisier data at low levels of IFNg. The legend says Figure 2B is a representative dataset of three. Given source data will need to be provided for all plots, it would be good to have the other two datasets provided in Supplementary figures so that the reader can make their own minds up about how similar the trends are.

5. Figure 1 shows schematics of how both pMHC titration and a time course of cytokine levels are needed to unpick effects of co-stimulation but all data figures only show the former; all time courses are plotted with derived values. Does re-slicing the raw datasets as time courses create plots similar to figure 1 schematics?

6. The receptor downregulation in Figure 2E is very pronounced. Is this downregulation dependent on TCR signaling though? I couldn't find a control to show whether pMHC binding was also required or if 4-1BBL or CD70 binding alone was sufficient to lose 4-1BB/CD27 expression. Given receptor downregulation is key to the later model, it would be good to have this information/control.

7. I couldn't see how the maximal rates of IFNg in Figure 2D were calculated from legend. If it's simply the gradient between points in Figure 2C (as legend implies), how can you still have 5 datapoints? Was this calculated per experiment or combined datasets, and if latter, how was error propagated?

8. Methods state that the expression level of transduced TCR was measured using tetramers by flow cytometry but no data for this is provided in manuscript. If the engineered TCR expression is very variable, it would be worthwhile showing this and discussing whether it might have any effect on results, as there is an underlying assumption that all transduced T cells will respond equivalently.

9. The efficient downregulation of CD27 on T cell activation is stated to explain its minimal effect in prolonging costimulatory effect, compared to 4-1BB. An interesting experiment then would be to co-transduce the T cells with a CD27-expressing lentivirus whose promoter is constitutive (like EF1a). If CD27 expression is resistant to signaling-induced decrease in expression, CD27 may become a very efficient costimulatory receptor, on par with 4-1BB. This is not an essential experiment, but could provide further direct evidence for their conclusions.

10. In the discussion, there is no real mention of the caveats to presenting T cell ligands immobilized to a hard plastic surface. I am not in any way devaluing the utility of the work, but it is nonetheless important for readers who are not familiar with this type of signaling to know that these ligands are normally on an apposing cell surface that might use other ways for signals to be integrated, through co-clustering, for instance. The authors themselves have recently shown that plate-bound ligands are not as efficient as those on presenting cells, so it would be worth re-iterating this point in the discussion of this manuscript.

11. The methods state that streptavidin plates were first coated with pMHC before the TNFSF ligands bound. The inference is that pMHC binding to the plate does not 'soak up' a significant proportion of the streptavidin binding sites and so the two binding

steps are independent. Is this the case? What if at high pMHC coating, the amount of potential CD70 binding is decreased compared to when equivalent concentration of CD70 is bound to wells with low levels of bound pMHC? Probably this is no issue, but it would be good to confirm that this is the case, through antibodies specific to CD70 or 4-1BBL, for instance.

Minor points

12. Line numbers would have helped the reviewing process.

13. First introductory paragraph seemed quite brief, especially for an audience that is unlikely to be conversant with general principles of T cell signaling.

14. Page 1, para 1: "...these receptors display a large variety". Better as "...display large variation"?

15. Page 1, para 1: "T cell responses" in final sentence isn't really defined. For broad reader, they are unlikely to know what these are. Perhaps link better with the next para which uses "functions" as synonym for "responses"

16. Page 1, para 2: Rationale not rational

17. Figure 2 legend title: Given whole figure describes IFNg, perhaps word cytokine should be replaced here with IFNg?

Point-by-point responses

Reviewer 3

We thank the reviewer for taking the time to read our work in detail and for providing constructive comments to improve the work.

1. Why were CD8+ T cells, and not CD4+ T cells used for these experiments, or even both? Co-stimulation is important for both T cell subsets. The differences between these subtypes were also not defined, which would be helpful for the general MSB reader. It would be good to explicitly state why the authors thought CD8+ T cells were the better choice for these experiments. Would they expect similar results for CD4+ T cells?

We do not necessarily expect similar results for CD4+ T cells, as the expression patterns of some TNFRSF members differ between CD8 and CD4+ T cells (Fig. S2) and it would be interesting to repeat the study with CD4+ T cells in the future. We selected CD8+ T cells for this study because, although cytokine production has been heavily studied for CD4+ T cells, there are comparatively fewer studies on CD8+ T cells, which is likely because it was initially thought that their role is confined to killing.

We could not easily introduce different T cell populations in the introduction without adding an entire paragraph. Instead, reference to CD4 T cells is made in the first results section and we have added another sentence to the discussion.

2. Following on from this, what state were activated/expanded CD8+ T cells in before plate-bound assays? How would this compare to naïve T cells, say? I think it is important to confirm what phenotype they had given that CTL, for instance, have minimal requirement for co-stimulation.

To generate our primary antigen-specific human CD8 T cells in sufficient numbers, we used a standard ex vivo transduction and expansion protocol that is used for adoptive cell therapy. These T cells are therefore no longer naïve and serve as in vitro models for effector/cytotoxic T cells as they can readily kill target cells and are able to rapidly secrete cytokines. Although killing and the initial cytokine burst have minimal co-stimulation requirements, cytokine production is markedly dependent on co-stimulation, as is evident from our data in Fig 2 for example. In contrast, naïve human CD8+ T cells do not appear to secrete cytokines even with co-stimulation (see Fig 2C in https://www.frontiersin.org/articles/10.3389/fimmu.2013.00455/full).

In the first result section we explain how we generate our T cells and explain that these are used in adoptive T cell therapies and that they also serve as in vitro models for human effector/cytotoxic T cells.

3. Page 1, Para 3: "Operational" needs to be defined explicitly within text, given its continued use when describing models later. The reference doesn't really help here.

We have added text to define it.

4. Page 4, para 2: It was quite hard to visualise the "burst of cytokine" when there isn't a time course to directly view this in Fig 2B. To me, it looked like for GITR and OX40 IFNg is increasing with time.

Maybe this is just noisier data at low levels of IFNg. The legend says Figure 2B is a representative dataset of three. Given source data will need to be provided for all plots, it would be good to have the other two datasets provided in Supplementary figures so that the reader can make their own minds up about how similar the trends are.

We now include all source data as excel files.

In Fig 2C/D we show Emax and the rate of change of Emax over time averaged over multiple repeats showing that GITR and OX40 have values that do not significantly differ from the condition without any TNFR ligand ('none').

5. Figure 1 shows schematics of how both pMHC titration and a time course of cytokine levels are needed to unpick effects of co-stimulation but all data figures only show the former; all time courses are plotted with derived values. Does re-slicing the raw datasets as time courses create plots similar to figure 1 schematics?

Yes. Below, we paste a time course from one repeat:

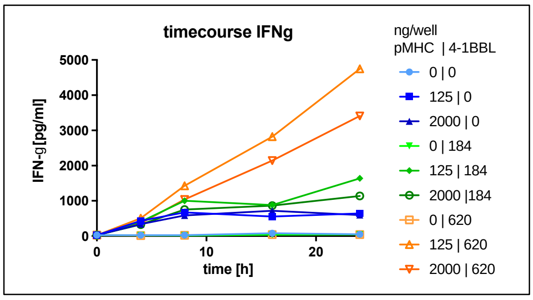

The problem we found of displaying all the data in this way was that there are a large number of curves (96 in total for each ligand condition) so it made the timecourse difficult to see (in the above we show a small subset of the data in the experiment). Instead, we plotted Emax over time (Fig 2C/D, Fig S1). We also found that Emax was less prone to noise and could be averaged across donors allowing us to more robustly display our data (instead of just representative repeats) and perform statistics.

6. The receptor downregulation in Figure 2E is very pronounced. Is this downregulation dependent on TCR signaling though? I couldn't find a control to show whether pMHC binding was also required or if 4-1BBL or CD70 binding alone was sufficient to lose 4-1BB/CD27 expression. Given receptor downregulation is key to the later model, it would be good to have this information/control.

No, we find that CD27 and 4-1BB can be downregulated without acute pMHC stimulus. In the case of 4-1BB, we do require pMHC to induce expression but if these T cells are then transferred to plates with 4-1BBL alone it is sufficient to induce downregulation.

We generate the following two plots to illustrate this:

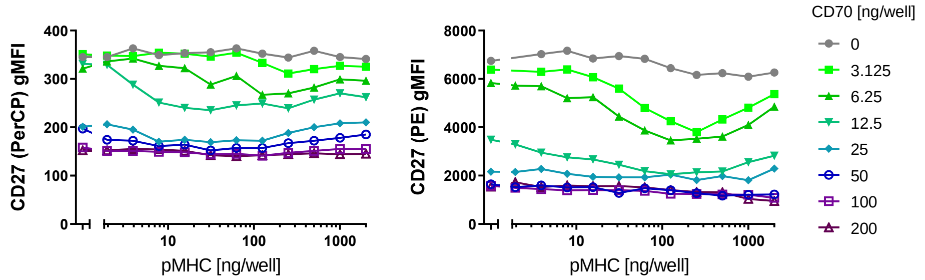

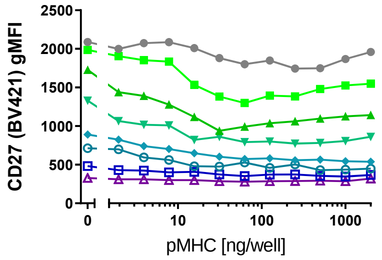

CD70 downregulates its receptor CD27 even in the absence of pMHC. Primary human CD8$^+$ T cells transduced with the c58/c61 TCR were stimulated for 4 hours with plate-immobilised pMHC and CD70 at the indicated doses. In three separate experimental repeats, surface CD27 was labelled with three different fluorophore-conjugated antibodies and quantified by flow cytometry.

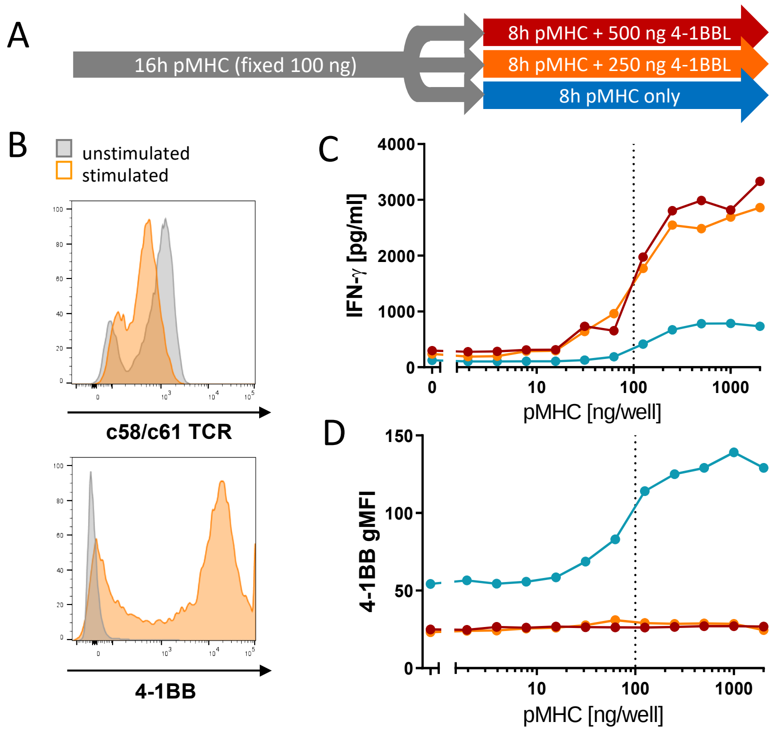

4-1BBL does not induce pMHC-independent cytokine responses but downregulates 4-1BB surface expression even in absence of pMHC.
Primary human CD8$^+$ T cells transduced with the c58/c61 TCR were uniformly stimulated for 16h with 100 ng/well pMHC in order to induce 4-1BB expression with minimal TCR downregulation. Cells were harvested, washed, and stimulated for a further 8h with pMHC and 4-1BBL at the doses indicated in **(A)**. **(B)** TCR and 4-1BB expression on cells before and after the 16h pre-stimulation from one representative experiment out of four. Surface c58/c61 TCR and 4-1BB were labelled with fluorescent high-affinity pMHC tetramers and anti-4-1BB antibodies, respectively, and quantified by flow cytometry. T cell response **(C)** and surface 4-1BB expression **(D)** during the secondary 8h stimulation from one representative experiment out of three. The production of IFN-γ into the culture medium supernatant was quantified by ELISA, and surface 4-1BB expression by flow cytometry.

We are conscious that we already have 9 supplementary figures and because this data is already included as part of the source data and that ligand-induced receptor downregulation is the 'null hypothesis' for how most receptors are downregulated, we would propose that these figures are not added to the manuscript.

7. I couldn't see how the maximal rates of IFNg in Figure 2D were calculated from legend. If it's simply the gradient between points in Figure 2C (as legend implies), how can you still have 5 datapoints? Was this calculated per experiment or combined datasets, and if latter, how was error propagated?

The rates were indeed calculated as gradients between points in Figure 2C. This was done per experiment and normalised to the mean rate at 4h without co-stimulation (i.e. errors are simply calculated from each independent repeat). The fifth 'data point' is the rate at t = 0, which we assumed is 0 because the T cells were resting before the assay. We have revised the caption to make this explicit. We now also explicitly show individual data points as per journal conventions.

8. Methods state that the expression level of transduced TCR was measured using tetramers by flow cytometry but no data for this is provided in manuscript. If the engineered TCR expression is very variable, it would be worthwhile showing this and discussing whether it might have any effect on results, as there is an underlying assumption that all transduced T cells will respond equivalently.

We found some variation in the transduction efficiency, and this could explain differences in the absolute level of cytokines produced by the population of T cells (i.e. higher transduction efficiencies lead to more T cells being able to respond). As a result, we were not able to directly average data across donors and instead, we first normalised the cytokine response within each donor before averaging. Given that we measured bulk cytokine secretion, we cannot directly assess whether all the transduced T cells within one experiment (within one donor's T cells) were behaving uniformly in terms of cytokine production. We have assessed at the single-cell level the expression of TCR and other surface molecules by flow cytometry (such as the activation markers CD69 and 4-1BB). Using this data, we can confirm a uniform population of activated T cells (e.g. all transduced cells expressed 4-1BB and CD69 at the maximum stimulus condition).

9. The efficient downregulation of CD27 on T cell activation is stated to explain its minimal effect in prolonging costimulatory effect, compared to 4-1BB. An interesting experiment then would be to co-transduce the T cells with a CD27-expressing lentivirus whose promoter is constitutive (like EF1a). If CD27 expression is resistant to signaling-induced decrease in expression, CD27 may become a very efficient costimulatory receptor, on par with 4-1BB. This is not an essential experiment, but could provide further direct evidence for their conclusions.

We agree that this experiment would support our conclusions and that it is not essential and given the timescale required to produce this data, we suggest that the manuscript is published in its current state. This idea can be used to increase efficacy of engineered T cells and we have included it in the discussion.

10. In the discussion, there is no real mention of the caveats to presenting T cell ligands immobilized to a hard plastic surface. I am not in any way devaluing the utility of the work, but it is nonetheless important for readers who are not familiar with this type of signaling to know that these ligands are

normally on an apposing cell surface that might use other ways for signals to be integrated, through co-clustering, for instance. The authors themselves have recently shown that plate-bound ligands are not as efficient as those on presenting cells, so it would be worth re-iterating this point in the discussion of this manuscript.

We have included a new paragraph on caveats of plate-presentation in the discussion. Briefly, mobility/clustering is important for signal integration where the mechanism requires proximity (e.g. when one receptor modifies another) but it is less clear whether it is important, or to what extent it is important, for TNFRSF that integrate signals more distally.

11. The methods state that streptavidin plates were first coated with pMHC before the TNFSF ligands bound. The inference is that pMHC binding to the plate does not 'soak up' a significant proportion of the streptavidin binding sites and so the two binding steps are independent. Is this the case? What if at high pMHC coating, the amount of potential CD70 binding is decreased compared to when equivalent concentration of CD70 is bound to wells with low levels of bound pMHC? Probably this is no issue, but it would be good to confirm that this is the case, through antibodies specific to CD70 or 4-1BBL, for instance.

We titrated the TNFRSF ligands and found that increasing their concentration could lead to increases in T cell responses (for CD70 and 4-1BBL), which supports the notion that the plate capacity was not reached even at high pMHC concentrations. This is consistent with the capture capacity of the plate (1.25×10^{-10} mol per well) being higher than the largest amount of pMHC that we used (2 ug/well or ~4×10^{-11} mol). The dose-response curves for pMHC show that even at high pMHC concentrations, CD70 and 4-1BBL continue to impact T cell responses. Therefore, if there was such a bias in the data it is likely to be modest and importantly, it would not impact our conclusions.

Minor points

12. Line numbers would have helped the reviewing process.

Apologies for this and we will include these next time.

13. First introductory paragraph seemed quite brief, especially for an audience that is unlikely to be conversant with general principles of T cell signaling.

This is now expanded.

14. Page 1, para 1: "...these receptors display a large variety". Better as "...display large variation"?

This is now revised.

15. Page 1, para 1: "T cell responses" in final sentence isn't really defined. For broad reader, they are unlikely to know what these are. Perhaps link better with the next para which uses "functions" as synonym for "responses"

This is now revised.

16. Page 1, para 2: Rationale not rational
This is now revised.

17. Figure 2 legend title: Given whole figure describes IFNg, perhaps word cytokine should be replaced here with IFNg?

This is now revised.

Thank you for sending us your revised manuscript. We have now heard back from the reviewer who was asked to evaluate your study. As you will see, the reviewer is satisfied with the modifications made and thinks that the study is now suitable for publication.

Before we can formally accept your manuscript, we would ask you to address the following editorial-level issues.

REFEREE REPORTS

Reviewer #3:

The authors have addressed my concerns well in the revised manuscript and their rebuttal. I am happy for this work to be published in its current form.

The authors have made all requested editorial changes.

Thank you again for sending us your revised manuscript. We are now satisfied with the modifications made and I am pleased to inform you that your paper has been accepted for publication.

EMBO PRESS

YOU MUST COMPLETE ALL CELLS WITH A PINK BACKGROUND

PLEASE NOTE THAT THIS CHECKLIST WILL BE PUBLISHED ALONGSIDE YOUR PAPER

Corresponding Author Name: Omer Dushek	
Journal Submitted to: EMBO Molecular Systems Biology	
Manuscript Number: MSB-2021-10560	

USEFUL LINKS FOR COMPLETING THIS FORM

http://www.antibodypedia.com
http://1degreebio.org
http://www.equator-network.org/reporting-guidelines/improving-bioscience-research-report
http://grants.nih.gov/grants/olaw/olaw.htm
http://www.mrc.ac.uk/Ourresearch/Ethicsresearchguidance/Useofanimals/index.htm
http://ClinicalTrials.gov
http://www.consort-statement.org
http://www.consort-statement.org/checklists/view/32-consort/66-title
http://www.equator-network.org/reporting-guidelines/reporting-recommendations-for-tumo
http://datadryad.org
http://figshare.com
http://www.ncbi.nlm.nih.gov/gap
http://www.ebi.ac.uk/ega
http://biomodels.net/
http://biomodels.net/miriam/
http://jjj.biochem.sun.ac.za
https://osp.od.nih.gov/biosafety-biosecurity-and-emerging-biotechnology/
http://www.selectagents.gov/

Reporting Checklist For Life Sciences Articles (Rev. June 2017)

This checklist is used to ensure good reporting standards and to improve the reproducibility of published results. These guidelines are consistent with the Principles and Guidelines for Reporting Preclinical Research issued by the NIH in 2014. Please follow the journal's authorship guidelines in preparing your manuscript.

A- Figures
1. Data
The data shown in figures should satisfy the following conditions:

- ➔ the data were obtained and processed according to the field's best practice and are presented to reflect the results of the experiments in an accurate and unbiased manner.
- ➔ figure panels include only data points, measurements or observations that can be compared to each other in a scientifically meaningful way.
- ➔ graphs include clearly labeled error bars for independent experiments and sample sizes. Unless justified, error bars should not be shown for technical replicates.
- ➔ if n< 5, the individual data points from each experiment should be plotted and any statistical test employed should be justified
- ➔ Source Data should be included to report the data underlying graphs. Please follow the guidelines set out in the author ship guidelines on Data Presentation.

2. Captions

Each figure caption should contain the following information, for each panel where they are relevant:

- ➔ a specification of the experimental system investigated (eg cell line, species name).
- ➔ the assay(s) and method(s) used to carry out the reported observations and measurements
- ➔ an explicit mention of the biological and chemical entity(ies) that are being measured.
- ➔ an explicit mention of the biological and chemical entity(ies) that are altered/varied/perturbed in a controlled manner.

- ➔ the exact sample size (n) for each experimental group/condition, given as a number, not a range;
- ➔ a description of the sample collection allowing the reader to understand whether the samples represent technical or biological replicates (including how many animals, litters, cultures, etc.).
- ➔ a statement of how many times the experiment shown was independently replicated in the laboratory.
- ➔ definitions of statistical methods and measures:
  - common tests, such as t-test (please specify whether paired vs. unpaired), simple χ2 tests, Wilcoxon and Mann-Whitney tests, can be unambiguously identified by name only, but more complex techniques should be described in the methods section;
  - are tests one-sided or two-sided?
  - are there adjustments for multiple comparisons?
  - exact statistical test results, e.g., P values = x but not P values < x;
  - definition of 'center values' as median or average;
  - definition of error bars as s.d. or s.e.m.

Any descriptions too long for the figure legend should be included in the methods section and/or with the source data.

In the pink boxes below, please ensure that the answers to the following questions are reported in the manuscript itself. Every question should be answered. If the question is not relevant to your research, please write NA (non applicable). We encourage you to include a specific subsection in the methods section for statistics, reagents, animal models and human subjects.

B- Statistics and general methods

Please fill out these boxes (Do not worry if you cannot see all your text once you press return)

1.a. How was the sample size chosen to ensure adequate power to detect a pre-specified effect size?	Defining a sample as T cells from independent human donors, we selected a sample size of 3. A power calculation was not possible because we did not have indication of the variability in the response to TNFRSF co-stimulation or which measures were to be compared. Statistics were only performed once all samples had been collected.
1.b. For animal studies, include a statement about sample size estimate even if no statistical methods were used.	NA
2. Describe inclusion/exclusion criteria if samples or animals were excluded from the analysis. Were the criteria pre-established?	Summary metrics (Emax, EC50) are obtained from dose-response curves but when the response is low, these estimates become unreliable. Metrics were not used if the value of Emax was below 50 pg/ml (see 'Data analysis' in Methods). This criteria was not pre-established and all data, including dose-response curves whose metrics were excluded are included in the source data.
3. Were any steps taken to minimize the effects of subjective bias when allocating animals/samples to treatment (e.g. randomization procedure)? If yes, please describe.	NA
For animal studies, include a statement about randomization even if no randomization was used.	NA
4.a. Were any steps taken to minimize the effects of subjective bias during group allocation or/and when assessing results (e.g. blinding of the investigator)? If yes please describe.	NA
4.b. For animal studies, include a statement about blinding even if no blinding was done	NA
5. For every figure, are statistical tests justified as appropriate?	Yes. Standard t-tests are used for pairwise comparisons and for multiple comparisons, we used ANOVA with a multiple test correction.
Do the data meet the assumptions of the tests (e.g., normal distribution)? Describe any methods used to assess it.	Statistics were performed on fitted Emax values across donors, which is expected to be normally distributed.
Is there an estimate of variation within each group of data?	No. There are <5 repeats per condition making it difficult to accurately estimate variation.

Is the variance similar between the groups that are being statistically compared?	As above, it cannot be directly assessed.

C- Reagents

6. To show that antibodies were profiled for use in the system under study (assay and species), provide a citation, catalog number and/or clone number, supplementary information or reference to an antibody validation profile. e.g., Antibodypedia (see link list at top right), 1DegreeBio (see link list at top right).	Included in structured Methods table
7. Identify the source of cell lines and report if they were recently authenticated (e.g., by STR profiling) and tested for mycoplasma contamination.	NA (primary cells were used to generate the data)

* for all hyperlinks, please see the table at the top right of the document

D- Animal Models

8. Report species, strain, gender, age of animals and genetic modification status where applicable. Please detail housing and husbandry conditions and the source of animals.	NA
9. For experiments involving live vertebrates, include a statement of compliance with ethical regulations and identify the committee(s) approving the experiments.	NA
10. We recommend consulting the ARRIVE guidelines (see link list at top right) (PLoS Biol. 8(6), e1000412, 2010) to ensure that other relevant aspects of animal studies are adequately reported. See author guidelines, under 'Reporting Guidelines'. See also: NIH (see link list at top right) and MRC (see link list at top right) recommendations. Please confirm compliance.	NA

E- Human Subjects

11. Identify the committee(s) approving the study protocol.	Medical Sciences Interdivisional Research Ethics Committee, University of Oxford
12. Include a statement confirming that informed consent was obtained from all subjects and that the experiments conformed to the principles set out in the WMA Declaration of Helsinki and the Department of Health and Human Services Belmont Report.	T cells were obtained from leukocyte cones purchased by the National Health Services (UK) or from healthy voltuneers according to ethics approved by the MSDIREC (R51997/RE001). We confirm that conset was obtained from all subjects.
13. For publication of patient photos, include a statement confirming that consent to publish was obtained.	NA
14. Report any restrictions on the availability (and/or on the use) of human data or samples.	No restrictions
15. Report the clinical trial registration number (at ClinicalTrials.gov or equivalent), where applicable.	NA
16. For phase II and III randomized controlled trials, please refer to the CONSORT flow diagram (see link list at top right) and submit the CONSORT checklist (see link list at top right) with your submission. See author guidelines, under 'Reporting Guidelines'. Please confirm you have submitted this list.	NA
17. For tumor marker prognostic studies, we recommend that you follow the REMARK reporting guidelines (see link list at top right). See author guidelines, under 'Reporting Guidelines'. Please confirm you have followed these guidelines.	NA

F- Data Accessibility

18: Provide a "Data Availability" section at the end of the Materials & Methods, listing the accession codes for data generated in this study and deposited in a public database (e.g. RNA-Seq data: Gene Expression Omnibus GSE39462, Proteomics data: PRIDE PXD000208 etc.) Please refer to our author guidelines for 'Data Deposition'.<br><br>Data deposition in a public repository is mandatory for:<br>a. Protein, DNA and RNA sequences<br>b. Macromolecular structures<br>c. Crystallographic data for small molecules<br>d. Functional genomics data<br>e. Proteomics and molecular interactions	No standardised data is produced in the manuscript and therefore, the data has not been deposited. Instead, and given it's small size, we included all source data as excel spreadsheets as a 'Supplementary Document'.
19. Deposition is strongly recommended for any datasets that are central and integral to the study; please consider the journal's data policy. If no structured public repository exists for a given data type, we encourage the provision of datasets in the manuscript as a Supplementary Document (see author guidelines under 'Expanded View' or in unstructured repositories such as Dryad (see link list at top right) or Figshare (see link list at top right).	All source data is included as a 'Supplementary Document'.
20. Access to human clinical and genomic datasets should be provided with as few restrictions as possible while respecting ethical obligations to the patients and relevant medical and legal issues. If practically possible and compatible with the individual consent agreement used in the study, such data should be deposited in one of the major public access-controlled repositories such as dbGAP (see link list at top right) or EGA (see link list at top right).	NA
21. Computational models that are central and integral to a study should be shared without restrictions and provided in a machine-readable form. The relevant accession numbers or links should be provided. When possible, standardized format (SBML, CellML) should be used instead of scripts (e.g. MATLAB). Authors are strongly encouraged to follow the MIRIAM guidelines (see link list at top right) and deposit their model in a public database such as Biomodels (see link list at top right) or JWS Online (see link list at top right). If computer source code is provided with the paper, it should be deposited in a public repository or included in supplementary information.	NA. The computational model is a set of 6 ODEs, which is sufficiently simple that we have provided it in its entirety in the Methods (''Mathematical modelling").

G- Dual use research of concern

22. Could your study fall under dual use research restrictions? Please check biosecurity documents (see link list at top right) and list of select agents and toxins (APHIS/CDC) (see link list at top right). According to our biosecurity guidelines, provide a statement only if it could.	NA
